# From Causal Networks to Adverse Outcome Pathways: A Developmental Neurotoxicity Case Study

**DOI:** 10.3389/ftox.2022.815754

**Published:** 2022-03-07

**Authors:** Živa Ramšak, Vid Modic, Roman A. Li, Colette vom Berg, Anze Zupanic

**Affiliations:** ^1^ Department of Biotechnology and Systems Biology, National Institute of Biology, Ljubljana, Slovenia; ^2^ Faculty of Chemistry and Chemical Technology, University of Ljubljana, Ljubljana, Slovenia; ^3^ Department of Environmental Toxicology, Eawag—Swiss Federal Institute of Aquatic Science and Technology, Duebendorf, Switzerland

**Keywords:** systems toxicology, adverse outcome pathway, causal network, toxicological network, neurotoxicity

## Abstract

The last decade has seen the adverse outcome pathways (AOP) framework become one of the most powerful tools in chemical risk assessment, but the development of new AOPs remains a slow and manually intensive process. Here, we present a faster approach for AOP generation, based on manually curated causal toxicological networks. As a case study, we took a recently published zebrafish developmental neurotoxicity network, which contains causally connected molecular events leading to neuropathologies, and developed two new adverse outcome pathways: Inhibition of Fyna (Src family tyrosine kinase A) leading to increased mortality via decreased eye size (AOP 399 on AOP-Wiki) and GSK3beta (Glycogen synthase kinase 3 beta) inactivation leading to increased mortality via defects in developing inner ear (AOP 410). The approach consists of an automatic separation of the toxicological network into candidate AOPs, filtering the AOPs according to available evidence and length as well as manual development of new AOPs and weight-of-evidence evaluation. The semiautomatic approach described here provides a new opportunity for fast and straightforward AOP development based on large network resources.

## Introduction

A decade ago, adverse outcome pathways (AOPs) (Ankley et al., 2010) have been put forward as a tool for organizing toxicological knowledge across different levels of biological organization, from the initial interaction of chemicals with the biological system (MIE = molecular initiating event) (Allen et al., 2014) to the individual and population level effects relevant for environmental risk assessment (AO = adverse outcome). The main idea of AOPs is collecting basic knowledge about biological systems and their chemical perturbations, and organizing it in easy to understand sequences of causally connected biological events (KE = key events; KER = key event relationship, i.e., how one KE is connected to another) (Villeneuve et al., 2014b) which allows risk assessors and toxicologists to identify chemicals likely to cause environmental harm. Additionally, through identification of knowledge gaps, AOPs inform future research and the development of novel biological assays that allow more specific *in vitro* chemical testing and reduction of animal testing (Groh et al., 2015). The long-term goal of the AOP framework is to develop AOPs that cover the whole space of chemically-induced biological perturbations and a complete set of assays required for comprehensive chemical risk assessment (Zupanic et al., 2018).

From their conception, several tools have been developed for easier development and management of AOPs. The AOP-wiki and the AOP knowledge base (https://aopwiki.org/; AOP-KB: https://aopkb.oecd.org/index.html) form an online portal hosted by the OECD that serves as the central AOP hub (Groh et al., 2015; Fay et al., 2017). The accepted core principles (Villeneuve et al., 2014a) and a handbook for AOP development (OECD iLibrary, 2021) serve as a standard that enables the development of high-quality and structurally similar AOPs, with comparable weight-of-evidence (WoE) evaluations. On top, several helper tools for AOP development and visualization have been made available to the community (e.g., http://datasciburgoon.github.io/aopxplorer). The AOPs in the AOP-wiki have been useful resources for quite diverse toxicological studies, e.g., to find chemicals likely to activate the AOPs (Jeong et al., 2019), to evaluate the hazard associated with specific chemicals and chemical groups (Carvaillo et al., 2019; Negi et al., 2021), to develop assays for *in vitro* assessment of mixture toxicity (Pistollato et al., 2020), to develop a new tiered testing approach for thyroid hormone disruptors (Knapen et al., 2020), to find the mechanisms of nanomaterial toxicity (Murugadoss et al., 2021) and to develop quantitative AOPs, mathematical models that can be used directly in chemical risk assessment (Margiotta-Casaluci et al., 2016; Doering et al., 2018; Perkins et al., 2019; Burgoon et al., 2020; Lillicrap et al., 2020).

As of August 2021, more than 400 AOPs and 5017 KEs have been developed [a large increase from 219 AOPs of April 2018 (Pollesch et al., 2019)], covering a range of species from nematodes to humans. However, these still cover only a very small part of the biological perturbations caused by chemical exposure and also only a few taxonomic groups. AOP development remains relatively slow, because each AOP requires searching for scientific literature, its manual curation, the formatting of the acquired knowledge into a user-friendly AOP and performing a WoE assessment (Vinken, 2013). To come closer to the final goal of a complete AOP space, the development of new AOPs needs to be accelerated.

There have been some attempts to do this already. The United States Environmental Protection Agency (EPA) has recently developed the Adverse Outcome Pathway database (AOP-DB), which can help with the annotation of AOP pathways under development, by connecting the information present on the AOP-wiki with various public resources [e.g., the NCBI gene, STRING (Szklarczyk et al., 2021) and Comparative Toxicogenomics Database (CTD) (Pittman et al., 2018; Davis et al., 2020)]. Other studies have tried to integrate publicly available resources [e.g., (ToxCast (Dix et al., 2007), CTD, Reactome (Jassal et al., 2020)] to develop AOP-like networks, which can serve as starting point for computationally predicted AOPs (cpAOPs). Several studies have used association rule mining to generate computationally predicted AOPs, mostly at the molecular level (Bell et al., 2016; Oki et al., 2016). Doktorova et al. have further developed a filtering approach for refining the molecular AOP-like networks using gene expression data from TG-Gates database (Igarashi et al., 2015) and manual curation to arrive at a putative AOP-network ending in the AO non-genotoxic induced hepatocellular carcinoma (Doktorova et al., 2020). The AOP-helpFinder tool uses text mining to find potential connections between key events (Jornod et al., 2021), while the computational pipeline developed by Jin et al. uses chemical-specific toxicogenomic data, pathway enrichment analysis and biomarker selection to develop putative cpAOPs (Jin et al., 2021).

Here we present a new approach for development of AOPs, based on a thus far AOP-untapped toxicological resource—causal toxicological networks (CTN) (Boué et al., 2015). CTNs are computational networks, which describe causally connected, mostly molecular, cellular and tissue events. We hypothesize that CTNs are especially appropriate as a starting resource for AOP development, as they are highly curated and all the connections in such networks are annotated by evidence. Compared to *de novo* AOP development, starting from such a network resource should decrease the time needed to search and curate scientific literature, but, since the CTNs are made from connected nodes and relationships between them, also make it easier to format the AOP into KE and KERs. The described approach is semiautomatic. It allows for automatic generation of a large number of candidate AOPs from a CTN, while the WoE evaluation and formatting of the evidence remains a manual process, as described by (Becker et al., 2015).

As a case study, we developed two developmental neurotoxicity AOPs. The choice of developmental neurotoxicity was made, because AOPs related to developmental neurotoxicity are still underrepresented in the AOP-wiki (Bal-Price et al., 2015; Knapen et al., 2015; Pistollato et al., 2020) and because of the recent activity in incorporation of AOP-based developmental neurotoxicity *in vitro* assays into chemical risk assessment (Sachana et al., 2021). This also allowed us to use our recently developed zebrafish causal developmental neurotoxicity network as starting point (Li et al., 2021). The new AOPs can be found on the AOP-wiki under Ids 399 and 410 (https://aopwiki.org/aops/399 and https://aopwiki.org/aops/410). Here, we present the details of the taken approach, the analysis of the differences between the CTNs and AOPs and the consequences for using the former as a source for the latter. We also provide tools and guidance for the development of AOPs from large network resources for the toxicological community.

## Methods

### From the Causal Toxicological Network to AOP Candidates

The zebrafish causal developmental neurotoxicity network, which is the basis of our AOP development, has been developed in an earlier publication, therefore we here only summarize the most relevant properties of the network for this study, and refer the reader to original publication for details (Li et al., 2021). CTN network development usually starts from a known adverse outcome of interest. For the network used as starting point for AOP development here, these were known zebrafish developmental nervous system-related pathologies (megalencephaly, microcephaly, microphthalmos, seizures, neurogenic inflammation, hydrocephalus). Then evidence for events leading to the pathologies are found in the scientific literature, specifically the evidence needs to be present in a peer reviewed publication. The scientific literature is queried using search engines such as Google Scholar, using keywords representing the specific molecular events. The evidence needs to show causality, i.e., a performed upstream perturbation leading to a measured downstream perturbation. With literature curation, upstream nodes directly affecting the pathology are added, then further upstream nodes directly connected to these nodes and so on, until no more evidence (in the form of causal experimentally validated relationships) is found in the literature. From the time of the first publication of this causal developmental neurotoxicity network, the network has been further extended and the version used as starting point in this paper (NTOX_BEL; Supplementary Material) features 515 nodes (ranging from protein activations and gene ontology terms to pathologies), with 682 edges between them. It was built based on evidence gathered from 90 zebrafish specific scientific articles and each edge in the network is annotated by the evidence behind it in the form of a BEL statement (Slater, 2014) and the reference from which the evidence is taken.

**FIGURE 1:**
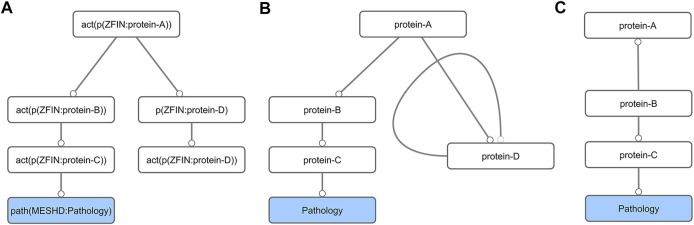
Schematic diagram of the network preparation procedure. First, the BEL network **(A)** is converted into its abstract form **(B)**. This simplification results in the formation of self-loops (protein-D), which are removed together with all nodes that do not lead to a pathology node **(C)**.

The original toxicological network (NTOX_BEL, Supplementary Material) contained nodes (molecular events) connected by edges (causal relationships between the nodes) and was written in Biological Expression Language (BEL) formalism. From the AOP development point of view it contained several non-necessary nodes (e.g., mRNA expression or protein activation of the same gene). To make it easier to manipulate with standard network analysis tools, we first generated a simplified abstracted network (NTOX_ABSTRACTED, Supplementary Material), i.e., the BEL formalism was simplified by removing unnecessary details from the BEL node and edge definitions (Figures 1A,1B; Supplementary Table S1). This abstracted network was then imported into Cytoscape [v3.8.0 (Shannon et al., 2003)] and further reduced by removing all self-loops. Finally, all nodes not connected to pathologies were removed by first selecting all nodes that had no outgoing connections, and then deleting anything that was not a pathology (Figures 1B,C). This step had to be repeated several times, until all the nodes in the network were connected to a pathology.

In addition to original annotations that the abstracted network inherited from the BEL formalism (e.g., references, experimental method), nodes were further manually curated and annotated. Each node in the network was first defined as a 1) coding or non-coding gene, the former further split into enzymes, transcription factors, receptors and transporters/channels, depending on the known or predicted function they perform (information taken from ZFIN and/or Gene Cards websites); 2) small molecule, which can be a metabolite (internal to the organism) or a chemical (externally added) (information taken from Chebi and/or KEGG databases; 3) biological process or a 4) pathology. All coding or non-coding genes were further annotated with gene identifiers (info taken from ZFIN, NCBI and/or ENSEMBL) and gene descriptions (ZFIN and UniProt). All nodes annotated as transporter/channel proteins, receptor proteins, enzymes and all targets of small molecules (metabolite or chemical) in the network were then marked as candidate MIE. These categories were chosen because they represent a vast majority of all MIEs currently in the AOP-wiki.

Next, all simple paths (defined as linear paths with every node present only once in a path) in the abstracted network starting at candidate MIEs and ending in nodes annotated as pathologies were found with the igraph package [v1.2.5 (Csardi and Nepusz, 2006)] in R [v4.0.2 (R Core Team, 2020)]. The resulting lists of simple paths were reformatted with a set of custom scripts (Supplementary Material) and form the basis for our choice of candidate AOPs to develop further.

From all candidate AOPs, we selected two that would be further developed into full feature AOPs, with additional condition that the new AOPs cannot be similar to any AOPs already present in the AOP-Wiki (on date: 10 Jan 2021) and that the new AOPs must feature more than two events above the cellular/tissues KE level. All the scripts for the network manipulations and instructions on how to use them can be found in the Supplementary material.

### Adverse Outcome Pathway Development and Weight-Of-Evidence Analysis

Although evidence on the KERs was already provided in the original toxicological network, they were based on a few articles, which were focused mostly on the causality between molecular events in zebrafish (so, mostly on biological plausibility of the KERs). This by itself is not enough for AOP development or WoE evaluation. We have therefore performed an additional search for evidence in the literature, seeking additional empirical evidence on dose and time concordance between connected KEs, additional evidence on the essentiality of the KEs and evidence on taxonomic applicability of the KERs. This search was performed using the search engines Google Scholar and Web of Science, using keywords relating to the key events. Additionally, papers citing the evidence already present in the CTN and scientific papers cited in the CTN evidence were used in the search. Also, as the candidate AOPs obtained from the CTN ended in a neuropathology, which conforms nicely to an organ/tissue level KE, we needed to find additional evidence to link this KE to AOs at the individual and population level (as per AOP definition). For all KEs and KERs in the two AOPs, we searched for new evidence through keyword based searches in GoogleScholar/Pubmed, but also took advantage of information already available in the AOP-wiki. We did this by connecting the KEs obtained from the CTN to KEs and KERs that have already been developed on the AOP-Wiki by other researchers. Whenever we made such a connection, we analyzed the evidence already present on the AOP-Wiki and contacted the authors of the KEs to ask for permission to add additional evidence to their KEs and KERs and to connect our AOP under development to them. After collecting all evidence, we performed a WoE evaluation.

The AOPs were developed based on the OECD Users’ handbook for developing and assessing AOPs (OECD, 2014). According to OECD instructions, the level of biological organization, taxonomic, life stage and sex applicability of the KE, as well as how the KE can be measured, were described. The description of KERs was based on their biological plausibility. WoE assessment of the AOPs was done through application of the modified Bradford-Hill criteria for biological plausibility and empirical evidence of KERs and essentiality of KEs (Becker et al., 2015). The WoE assessments were conducted for the individual KERs and KEs of the AOP as well as for the overall AOP. The level of support for the KEs and KERs was classified as high, moderate or low, using the criteria outlined in the OECD Users’ handbook (OECD, 2014).

## Results

### From Causal Networks to Candidate Adverse Outcome Pathways

**FIGURE 2:**
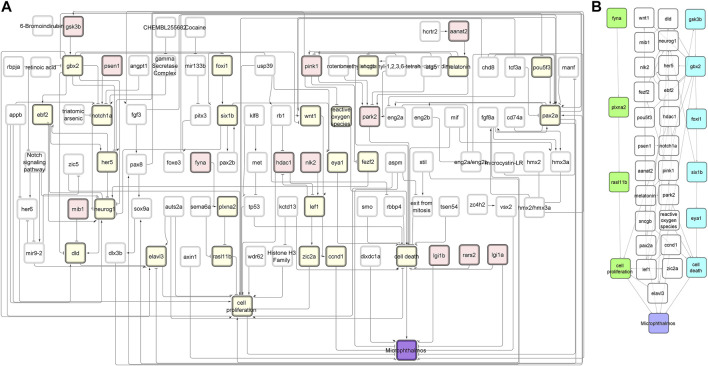
NTOX network. **(A)** The reduced NTOX network (125 nodes, 212 connections) where the start nodes are enzymes (red) and the final node is microphthalmos (purple). All other nodes that are part of the candidate AOPs are marked in yellow. **(B)** The subnetwork of the candidate AOPs that end with microphthalmos. The two candidate AOPs that were further developed into AOPs are marked in green (AOP 399) and blue (AOP 410).

The first step of the network analysis was cleaning the original toxicological network (NTOX_BEL), i.e., adding and removing annotations and joining network nodes representing the same genes (NTOX_ABSTRACTED), then removing self-loops and unconnected components and finally removing all nodes that are not directly connected to pathologies. Table 1 shows how the size of the network changed with each manipulation step, going from 515 nodes and 682 edges in the original network to 125 nodes and 212 edges after the final step (Figure 2, Supplementary material)

**TABLE 1:** Zebrafish causal developmental network reduction through the different network manipulation steps described in Materials and Methods.

	Number of nodes	Number of edges
NTOX_BEL (original)	515	682
NTOX_ABSTRACTED	297	678
After self-loop removal	297	459
After removing nodes not leading to pathologies*	125	212
Putative MIEs		
B: small molecule targets	17	—
C1: transporters and channels	4	—
C2: receptors	8	—
C3: enzymes	12	—
pathologies	6	—

The network was separated into individual simple paths from candidate MIE nodes to pathology nodes. In total, 391 simple paths were found to exist and be reachable between the candidate MIE and the pathologies (Table 2). We call these paths candidate AOP from here on, although they are not yet connected to an individual or population level AO. When connecting all simple paths from individual type of MIE to each pathology into subnetworks, the microphthalmos and microcephaly subnetworks were the largest, with more than 100 simple paths leading to each of them, none were found for megalencephaly, while only a couple of simple paths leading to the other four pathologies (Table 2). To reduce the number of simple paths further, we removed all those paths that had less than two molecular level KEs. We decided to do this, because the strength of the original toxicological network lies at the molecular level, therefore it makes more sense to use the toxicological network to develop AOPs with a strong molecular KE component. This reduced the total number of candidate AOPs to 329 (Table 2).

**TABLE 2:** The number of simple paths (candidate AOPs) found for each candidate MIE node type/pathology combination.

Candidate MIEs	Hydrocephalus	Microcephaly	Microphthalmos	Neurogenic inflammation	Seizures
Not filtered					
B	—	97	95	—	2
C1	4	3	2	—	—
C2	2	31	29	1	—
C3	1	60	60	1	3
Size filtered					
B	—	87	87	—	—
C1	—	—		—	—
C2	—	25	25	1	—
C3	—	52	52	—	—

### Adverse Outcome Pathway Development

Since even after network manipulations described in the previous section, there were still many candidate AOPs left with high-quality evidence, we were not able to come to a choice of best candidate AOPs based on the list alone. We therefore arbitrarily selected two candidate AOPs ending in microphthalmos (reduced eye size), which matched the scientific interest of the authors in the impairment of sensory organ development.

As has become custom in publications describing AOPs, the following AOP descriptions include the relevant chemical initiators of the AOPs, the KEs and KERs, the associated WoE for each, and the methods available for KE measurement (LaLone et al., 2017). As the focus of this paper is to evaluate WoE associated with AOPs generated from CTNs, biological plausibility of the KERs, essentiality of the KEs and empirical evidence as well as lack thereof are all discussed in detail.

Contrary to the authors of the earliest adverse outcome pathways in the AOP-Wiki, who had to describe all the KEs and KERs anew, some of the KEs and KERs of the new AOPs developed in this study were already described in the AOP-Wiki. We were thus able to take advantage of these descriptions, and only added new evidence when necessary to fit better with the zebrafish focus of our work. In this paper, for all KEs and KERs that are part of the new AOPs described here, but have been started by other researchers, we provide url links to the AOP-wiki. It is also worth mentioning that the AOP description in the following pages present a detailed, but still partly summarized description of all the information available on the AOP-Wiki. For a complete description, the reader is therefore invited to look up the AOPs 399 and 410 in whole on the AOP-Wiki.

### Adverse Outcome Pathway Description

#### Inhibition of Fyna Leading to Increased Mortality *via* Decreased Eye Size (Microphthalmos) [AOP 399] https://aopwiki.org/aops/399

**FIGURE 3:**
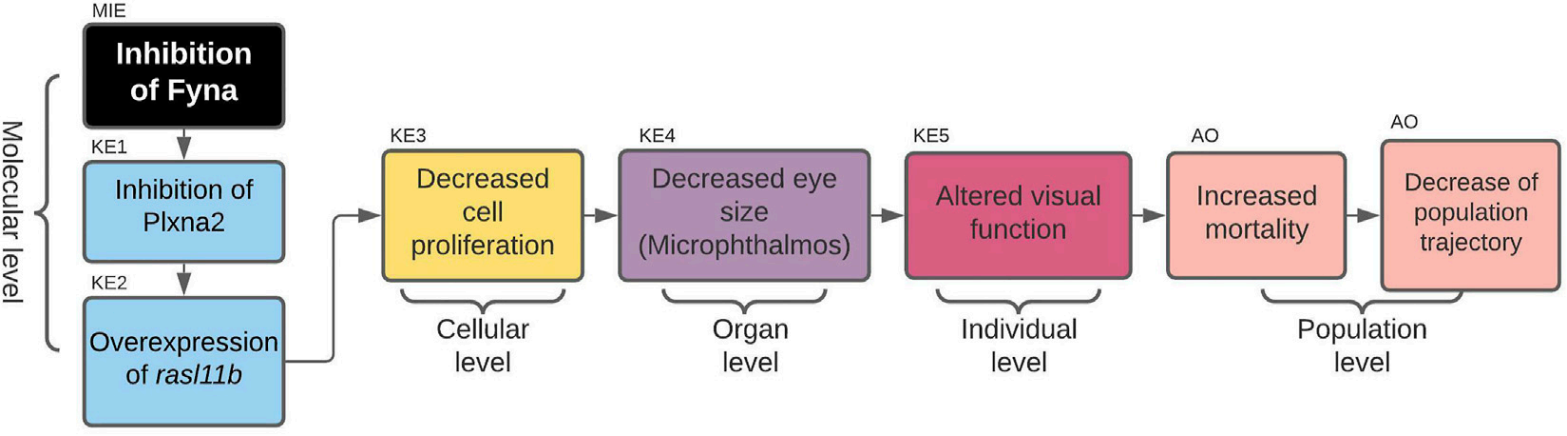
AOP 399. Inhibition of Fyna leads to increased mortality via decreased eye size (microphthalmos).

The first AOP to be developed was the »Inhibition of Fyna leading to increased mortality via decreased eye size (Microphthalmos)«, which can be found in the AOP-Wiki under AOP 399. The simple path that served as a basis for this AOP was found in the subnetwork containing all simple paths starting with enzymes and ending with microphthalmos (Figure 2A). The starting point of the development of AOP 399 was the MIE: inhibition of Fyna and the ending point the organ level KE: Decreased eye size. Since the original toxicological network did not feature any nodes interpretable as adverse outcomes at the individual or population level, we had to find additional evidence leading from KE: decreased eye size downstream to make a complete AOP. The final AOP 399 is visualized in Figure 3.

##### Chemical Initiators

Src family tyrosine kinase A (Fyna) is known to be inhibited by the herbal plant secondary metabolite rosmarinic acid (Jelić et al., 2007), by the microbial alkaloid staurosporine (Kinoshita et al., 2006) and saracatinib, a dual src family kinases (SFK) inhibitor, considered as a candidate drug for thyroid cancer and Alzheimer’s disease (Green et al., 2009). None of these chemical initiators is currently expected to be found in the environment in concentrations causing immediate concern. However, as FYN (the human ortholog of Fyna) is a potential target for medical treatments, drugs targeting FYN have the potential to become environmentally relevant pollutants.

##### MIE: Inhibition of Fyna

Fyna is a member of non-receptor tyrosine kinases, a group of enzymes that, after activation, transmit signals from several surface receptors to target proteins by phosphorylating tyrosine residues (Sen and Johnson, 2011). In zebrafish, Fyna was shown to be involved in several neurodevelopmental processes, such as gastrulation, adherens junction maintenance and regulation of axon growth, and to be required for normal brain development (Sharma et al., 2005). The human ortholog of *fyna* (FYN) is implicated in Alzheimer’s disease and schizophrenia (Challa and Chatti, 2013).

Essentiality—no experimental evidence found.

##### KER1: Inhibition of Fyna Leads to Inhibition of Plxna2

In zebrafish, mouse and human cell lines, Fyna was shown to constitutively associate with and phosphorylate the intracellular region of Plexin A1a (Plxna1) and Plxna2 (Sasaki et al., 2002; St Clair et al., 2018). To show that FYN induces PLXNA2 tyrosine phosphorylation, HEK293 cells were transfected with expression plasmids encoding *PLXNA2* and either Fyn wild-type (WT) or a kinase dead (KD) point mutant. PLXNA2 showed prominent tyrosine phosphorylation when FYN WT was expressed and this phosphorylation was absent when FYN KD was expressed (St Clair et al., 2018). In zebrafish, it was found that Fyna-dependent Plxna2 phosphorylation is critical for zebrafish eye development *in vivo*. Antisense oligonucleotides targeted to *plxna2* resulted in decreased proliferation of retinal precursor cells (Emerson et al., 2017).

Biological plausibility—moderate. Relationship evidence of phosphorylating activity of Fyna kinase and consequent changes in activity of Plxna2 is sufficiently researched and described for vertebrate models (Sasaki et al., 2002; Franco and Tamagnone, 2008; St. Clair et al., 2018). Fyna phosphorylation of intracellular domains of Plxna2 is crucial for translation of Plxna2 signals and normal development of eyes in zebrafish embryos (St. Clair et al., 2018). For better understanding of the relationship, data on the chemical inhibition of zebrafish Fyna kinase, its effect on phosphorylation of Plxna2 tyrosine residues, and further signaling is needed.

##### KE1: Inhibition of Plxna2

Plexins (Plxns) are receptors encoded by the members of the *plexin* gene family. They are the primary transducers of vertebrate Semaphorin (Sema) signals (Tamagnone et al., 1999). Semaphorins are members of a large gene family of secreted and membrane-anchored proteins, initially characterized as axon guidance factors (Nakamura et al., 2000). The receptors belonging to the Plexin family function as Semaphorin receptors (Neufeld and Kessler, 2008). Semas were initially discovered with respect to their role as repulsive guidance cues for migrating axons, although it is now appreciated that they have much broader roles in development. Semas and Plxns have tissue-specific expression patterns, and many Semas can signal through multiple Plxn family members (Luo et al., 1993). Plxna2 is involved in optic vesicle formation, and is predicted to localize to integral components of the plasma membrane and the Semaphorin receptor complex. It has been shown to be critical to zebrafish eye development (Ebert et al., 2014; St Clair et al., 2018).

Essentiality—moderate. Inhibition of Fyna kinase inhibits the function of plxna2 because of the lack of tyrosine residue phosphorylation in plxna2 and causes the reduction of zebrafish eye size. The same was also achieved by mutating the tyrosine residues of plxna2 to phenylalanine. However, the reduced eye size phenotype can be rescued by adding wild-type human PLXNA2 mRNA to the larva, indicating essentiality of plxna2 for this AOP (St. Clair et al., 2018).

##### KER2: Inhibition of Plxna2 Leads to Overexpression of rasl11b

RAS-like, family 11, member B (Rasl11b) is negatively regulated downstream of Sema6a/Plxna2 (semaphoring 6A) signaling and when overexpressed, decreases retinal precursor cells proliferation and eye size (Emerson et al., 2017). Rasl11b was found to be a target of Plxna2 regulation in a microarray study using zebrafish embryos deficient in either *sema6a* or *plxna2*. Rasl11b had a 2.18 log-fold change in expression (logFC) in sema6a morphants and a 1.58 logFC in plxna2 morphants. The microarray results were confirmed in independent experiments, using RT-PCR as readout. Further characterization *rasl11b* revealed its role in regulating retinal progenitor cell (RPC) proliferation.

Biological plausibility—low. Current knowledge of interactions between Plxna2 and Rasl11b are consistent in scientific literature, but there are one or more missing molecular links between the KE1 and KE2.

##### KE2: Overexpression of rasl11b

Rasl11b is a member of the small GTPase protein family with a high degree of similarity to RAS proteins (Stolle et al., 2007). Rasl11b is highly conserved among vertebrates, sharing on average 94% homology with its mammalian orthologs. In zebrafish, it has been shown to be involved in mesendoderm development (Pézeron et al., 2008). Ras proteins are otherwise known to be involved in the mitogen-activated protein kinase (MAPK) pathway (Emerson et al., 2017).

Essentiality—no experimental evidence found.

##### KER3: Overexpression of rasl11b Leads to Decreased Cell Proliferation

It is hypothesized that Rasl11b acts as a negative regulator of MAPK by outcompeting Ras for its effectors such as Raf, leading to decreased cell proliferation. In zebrafish, it has been shown that after overexpression of *rasl11b*, the G0/G1 phase cell cycle arrest is induced and the proliferation of retinal progenitor cells (RPCs) is inhibited in a dose-dependent manner (Emerson et al., 2017).

Biological plausibility—high. Impact of Rasl11b on overall proliferation is scientifically well supported, and the evidence from studies on zebrafish (Emerson et al., 2017) and human cancer cells (He et al., 2018) is consistent.

##### KE3: Decreased Cell Proliferation (https://aopwiki.org/events/1821)

Essentiality—no experimental evidence found.

KER4: Decreased cell proliferation leads to decreased eye sizeIn the same overexpression experiment described above, it was also determined that overexpression of *rasl11b* resulted in smaller eyes. It was confirmed through morpholino knockdown, that Sema6a/Plxna2 signaling regulates proliferation and cohesive migration of RPCs in developing optic vesicles in zebrafish (Emerson et al., 2017).

Biological plausibility—high. There is extensive evidence linking decreased proliferation of retinal precursor cells to decreased eye size in vertebrates. For the relationship support there are numerous studies of the effect of knockdown/knockout genes (*rasl11b*, *rx3*, *vsx2*) in vertebrates involved in the proliferation of retinal progenitor cells, which leads to reduced size of the eye or even absence of it (Burmeister et al., 1996; Kennedy et al., 2004; Graw, 2010; Zagozewski et al., 2014; Emerson et al., 2017). Also there are some studies of stressor (DEAB, citral) effects to support the relationship (Marsh-Armstrong et al., 1994; Le et al., 2012)

##### KE4: Decreased Eye Size (https://aopwiki.org/events/1878)

Essentiality—no experimental evidence found.

##### KER5: Decreased Eye Size Leads to Altered Visual Function (https://aopwiki.org/relationships/2377)

Biological plausibility - high. The impact of changes to the eye size on altered visual function has been well established and researched. Although the change in eye size is not the only indicator of impaired eye function, it is a good indicator of changes in eye development that can lead to impaired eye function.

##### KE5: Altered Visual Function (https://aopwiki.org/events/1643)

Essentiality—no experimental evidence found.

##### KER6: Altered Visual Function Leads to Increased Mortality (https://aopwiki.org/relationships/2375)

Biological plausibility—high. The link between impaired visual function and increased mortality is well accepted and proven. Evidence has been obtained in fish that have been exposed during development to various disruptors that affect eye development.

##### AO: Increased Mortality (https://aopwiki.org/events/351)

Essentiality—high. Mortality on individual level must increase for population trajectory to decrease.

##### Overall Assessment of the AOP 399

An overall assessment of this AOP shows that there is moderate biological plausibility to support a qualitative link between the Fyna kinase inhibition to the KE5 of altered visual function and high evidence linking KE5 to decreased population trajectory. Biological plausibility is considered moderate because there is ample evidence from gain- and loss- of function experiments and knock out animal models that support the relationships between key events and are consistent with current biological knowledge. A score of high in this respect would require further evidence for chemical inhibition or experimental downregulation of zebrafish Fyna kinase and direct or more extensive evidence linking Plxna2 inhibition to rasl11b overexpression. No empirical evidence in the form of dose response, time concordance and incidence concordance was found for the KERs of the AOP, and only little evidence for the essentiality of the KEs. The summarized information on KER biological plausibility, KE essentiality and KE observation methods of AOP 399 can be found in Tables 3–5.

**TABLE 3:** Assessed biological plausibility of the KERs in AOPs 399 and 410.

	KER	Biological plausibility
AOP 399	KER1: Inhibition of Fyna leads to inhibition of Plxna2	Moderate: Extensive understanding of Fyna phosphorylating activity and consequent changes in Plxna2 signalization, but there is currently no data on chemical inhibition of zebrafish Fyna kinase
	KER2: Inhibition of Plxna2 leads to overexpression of *rasl11b*	Low: There is missing direct evidence for the relationship and poor functional and structural understanding of interactions
	KER3: Overexpression of *rasl11b* leads decreased cell proliferation	High: Impact of Rasl11b on cell proliferation is well understood across different taxonomic groups
	KER4: Decreased cell proliferation leads to decreased eye size	High: Extensive understanding that decreased proliferation of RPCs leads to decreased eye size
	KER5: Decreased eye size leads to altered Visual function	High: Extensive understanding that changes in eye size greatly effect visual function
	KER6: Altered visual function leads to increased mortality	High: Extensive understanding that defective visual function greatly increases the chance of death due to various factors
	KER7: Increased mortality leads to decrease of population trajectory	High: Extensive understanding that increased mortality on individual level decreases population trajectory
AOP 410	KER1: Gsk3b inactivation leads to repression of *gbx2* expression	High: There is extensive evidence linking inhibition of Gsk3b to activation of canonical Wnt pathway for which Gbx2 is representative marker
	KER2: Repression of *gbx2* expression leads to increased *foxi1* expression	Moderate: Extensive evidence that Gbx2 represses many developmental regulatory genes such as *foxi1*, but multifunctional nature of Gbx2 is still unknown
	KER3: Increased*foxi1* expression leads to increased *six1b* expression	Low: Relationship was confirmed with loss-of-function experiment, but the connection could be secondary to the overall absence of otic placode
	KER4: Increased *six1b* expression leads to inhibited *eya1* expression	Low: Mutual regulation and interactions of both entities have not yet been well researched and described. Inconsistencies in zebrafish and mouse models
	KER5: Inhibited *eya1* expression leads to increased cell death	High: Extensive evidence of relationship in vertebrate models
	KER6: Increased cell death leads to altered inner ear development	High: Extensive understanding that inner ear development depends on correct regulation of cell death in precursor cells and tissues
	KER7: Altered inner ear development leads to reduced hearing	High: Extensive understanding of defects in the development of inner ear and outcomes suggestive of deafness
	KER8: Reduced hearing leads to increased mortality	High: Extensive understanding that defective hearing decreases survival in natural setting
	KER9: Increased mortality leads to decrease of population trajectory	High: Extensive understanding that increased mortality on individual level decreases population trajectory

**TABLE 4:** Assessed essentiality of the KEs in AOPs 399 and 410.

	Key event	Support for essentiality	References
AOP 399	MIE: Inhibition of Fyna	No experimental evidence of essentiality	—
	KE1: Inhibition of Plxna2	Moderate: reduced eye size phenotype can be rescued by plxna2 activation	St Clair et al. (2018)
	KE2: Overexpression of *rasl11b*	No experimental evidence of essentiality	—
	KE3: Decreased cell proliferation	No experimental evidence of essentiality	—
	KE4: Decreased eye size	No experimental evidence of essentiality	—
	KE5: Altered visual function	No experimental evidence of essentiality	—
	AO: Increased mortality	High: Inability to perceive the environment leads to increase in mortality	Dehnert et al. (2019); Besson et al. (2020)
	AO: Decrease of population trajectory	High: decrease in population trajectory is an imminent result of increased mortality	Rearick et al. (2018)
AOP 410	MIE: Gsk3b inactivation	No experimental evidence of essentiality	—
	KE1: Repression of *gbx2* expression	No experimental evidence of essentiality	—
	KE2: Increased *foxi1* expression	High: When *foxi1* is knock down no expression of *six1b* is detected in otocyst	Bricaud and Collazo, (2006)
	KE3: Increased *six1b* expression	Moderate: *Six1b* gain/loss-of-function experiment results indicate that in both cases normal development of inner ear is affected (KE5)	Bricaud and Collazo, (2006)
	KE4: Inhibited *eya1* expression	No experimental evidence of essentiality	—
	KE5: Increased cell death	High: One of key players in normal development of sensory organs (KE6)	Whitfield et al. (2002); Kozlowski et al. (2005)
	KE6: Altered inner ear development	No experimental evidence of essentiality	—
	KE7:Reduced hearing	Moderate: One of the factors that are responsible for higher rate of mortality in fish (KE8)	Kasumyan, (2009)
	AO: Increased mortality	High: Inability to perceive the environment leads to increase in mortality	Besson et al. (2020)
	AO: Decrease of population trajectory	High: decrease in population trajectory is an imminent result of increased mortality	Rearick et al. (2018)

**TABLE 5:** Measurement methods available for the KEs in AOPs 399 and 410.

	Key event	Methods of observation, examples
AOP 399	MIE: Inhibition of Fyna	• ELISA using anti tyrosine phosphate antibody Jelić et al. (2007)
		• Adp-Glo ^TM^ Bioluminescent and homogeneous ADP monitoring assay Zegzouti et al. (2009)
	KE1: Inhibition of Plxna2	• Phosphorylation changes can be detected directly using western blot St Clair et al. (2018) and indirectly using ELISA. Several antibodies available commercially
	KE2: Overexpression of *rasl11b*	• Reverse transcription polymerase chain reaction (RT-PCR) (Wong and Medrano, 2005)
	KE3: Decreased cell proliferation	• Measuring DNA synthesis in dividing cells with BrdU and EdU assay Salic and Mitchison (2008); Mead and Lefebvre (2014)
	KE4: Decreased eye size	• Ocular biometry Kang and Wildsoet (2016)
		• Measurement of relative eye size: larger corneal diameters relative to the axial length or larger eye diameter relative to body length Hall (2008); Baumann et al. (2016) determined by morphological analysis with microscopy or analysis of digital images
	KE5: Altered visual function	• Assaying opto kinetic response , swimming activity, light preference Baumann et al. (2016)
		• Diverse mobility assay: tracking assays, phototactic swimming activity assay Gao et al. (2015); Baumann et al. (2016)
		• Electroretinogram measurements Fleisch et al. (2008)
	AO: Increased mortality	• Measured by recording increase in deaths in study setup compared to control
	AO: Decrease of population trajectory	• Estimation by population modeling based on measurements of vital rates or reasonable surrogates measured in laboratory setup Miller and Ankley (2004).
AOP 410	MIE: Gsk3b inactivation	• Wnt/beta-catenin activity assay Zhou et al. (2002); Naujok et al. (2014)
		• Immunoprecipitation of total cellular GSK3 Cole and Sutherland (2008)
		• Immunoblot using antibodies to phospho-Ser9 Cole and Sutherland (2008)
	KE1: Repression of *gbx2* expression	• Reverse transcription polymerase chain reaction (RT-PCR) Wong and Medrano (2005)
	KE2: Increased *foxi1* expression	• Reverse transcription polymerase chain reaction (RT-PCR) Wong and Medrano (2005)
	KE3: Increased *six1b* expression	• Reverse transcription polymerase chain reaction (RT-PCR) Wong and Medrano (2005)
	KE4: Inhibited *eya1* expression	• Reverse transcription polymerase chain reaction (RT-PCR) Wong and Medrano (2005)
	KE5: Increased cell death	• Staining cell sample with trypan blue, TUNEL technique, detection of nuclear condensation, detection of DNA fragmentation. Assays are described in detail in Crowley et al. (2016)
	KE6: Altered inner ear development	• Direct observation of internal anatomic structures Whitfield (2002)
	KE7: Reduced hearing	• Startle response Colwill and Creton (2011)
		• Comparison of swimming patterns with wild-type fish Nicolson et al. (1998)
		• High throughput behavioral test for detecting auditory response Bang et al. (2002)
		• Microphonic potential recordings Yao et al. (2016)
	AO: Increased mortality	• Measured by recording increase in deaths in study setup compared to control
	AO: decrease of population trajectory	• Estimation by population modeling based on measurements of vital rates or reasonable surrogates measured in laboratory setup Miller and Ankley (2004)

#### Gsk3beta Inactivation Leads to Defects in Developing Inner Ear and Consequently to Increased Mortality [AOP 410] https://aopwiki.org/aops/410

The linear path from which we started to develop our second AOP (AOP 410) was located in the subnetwork that contained all linear paths that started with enzymes and led to microphthalmia (reduced eye size) (Figure 2). By comparing the above sentence to the title of the AOP, it is going to be immediately clear to the reader that the development of AOP 410 did not go as smoothly as for AOP 399 and we have ended up developing a different AOP than was initially planned.

**FIGURE 4:**
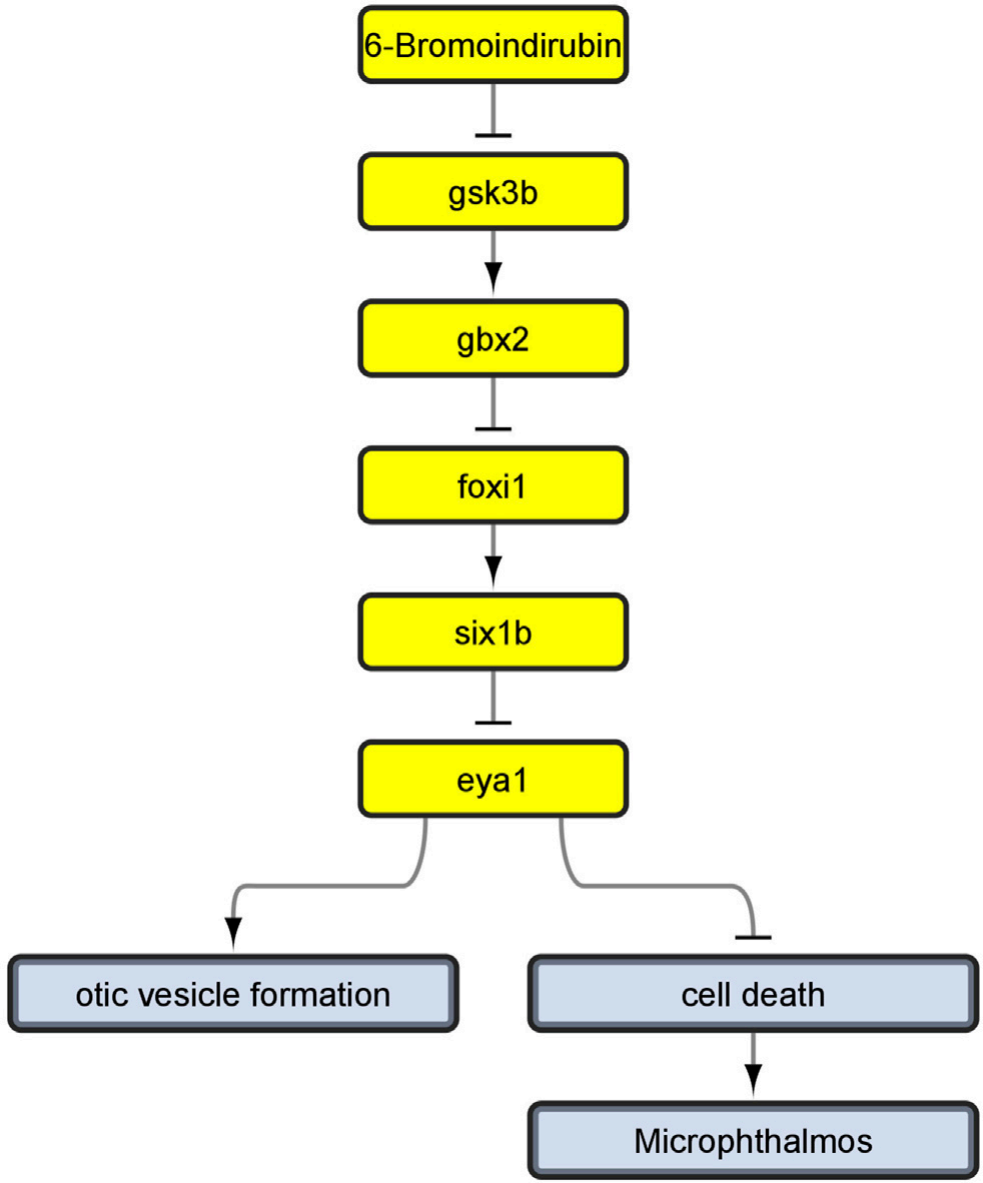
Linear path which we developed into AOP 410 and its connection to otic vesicle formation node before removing non-pathology-ends. Yellow nodes represent linear path upstream of cell death.

**FIGURE 5:**
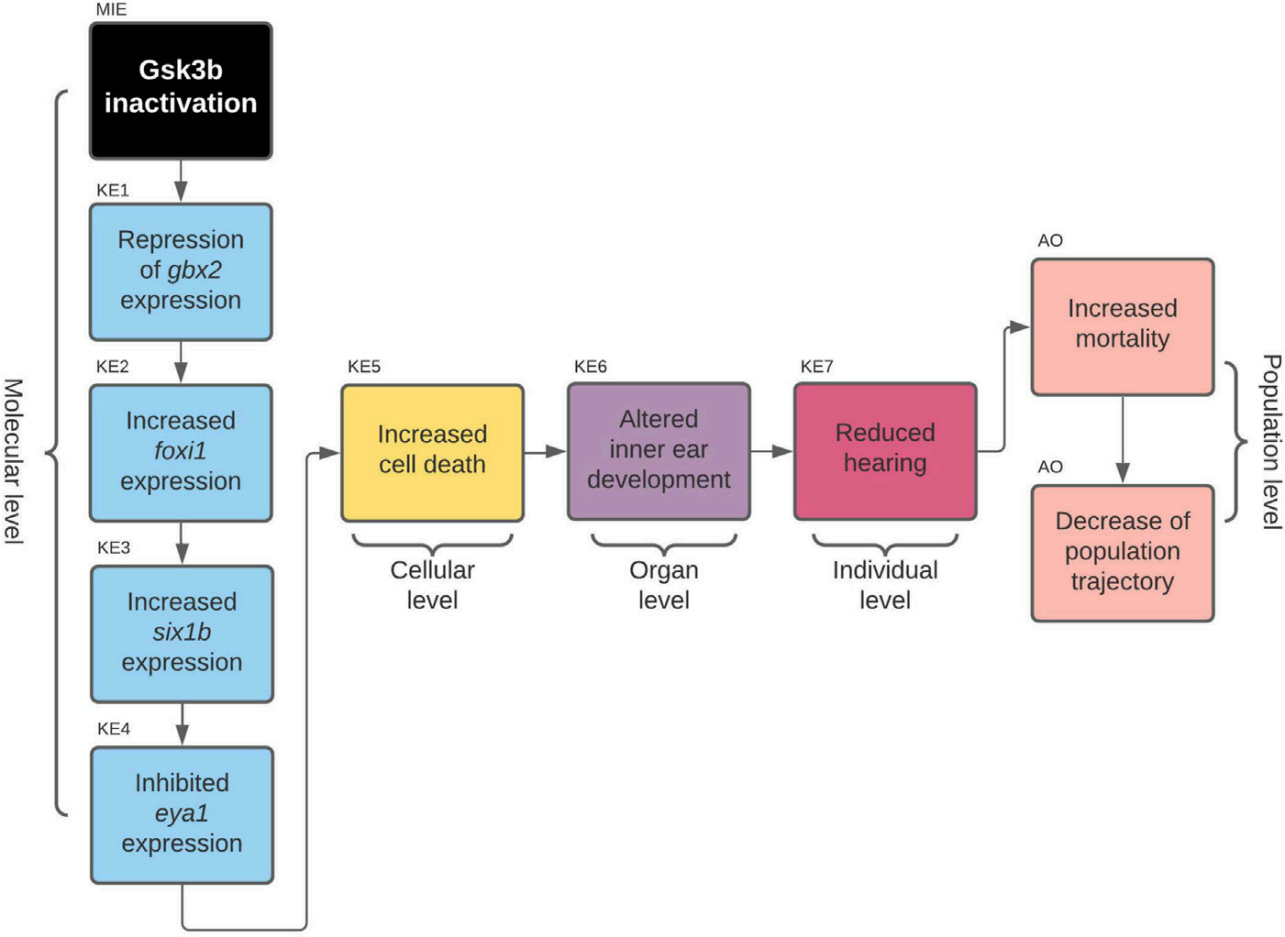
AOP 410. Gsk3beta inactivation leads to increased mortality *via* defects in development of the inner ear.

This is because, during the review of the evidence for AOP 410, we encountered a problem: Evidence linking cell death to microphthalmos was inconsistent with evidence for upstream KERs. Namely, all additional evidence we have found for upstream KERs referred to perturbations during inner ear development (Solomon et al., 2003; Kozlowski et al., 2005; Bricaud and Collazo, 2006) while the KER linking cell death and microphthalmia in the original CTN referred to cell death in developing eye (Tsai et al., 2015; Schultz et al., 2018). Since the evidence did not relate to a common pathology, we reversed back to the full version of the toxicological network to see if any nodes have been lost while applying network filters. We found that the selected pathway upstream of cell death also led to node otic vesicle formation (Figure 4) which got removed during filtering, since it did not lead to any pathologies that was selected to be part of the original neurotoxicity network. As the evidence throughout the pathway was therefore more consistent for KE:altered inner ear development, we decided to switch our focus to the alternate AOP. The final developed AOP 410 is visualized in Figure 5.

#### Chemical Initiators

Glycogen synthase kinase three beta (Gsk3b) has been shown to be inactivated by 6-bromoindirubin-3′-oxime (BIO), a selective GSK3 inhibitor that activates Wnt signaling, su5402, a growth factor receptor inhibitor, and retinoic acid, a metabolite involved in embryonic development. This regulation was shown in mouse (Sato et al., 2004) and zebrafish embryos (Wang et al., 2018). In the zebrafish experiments, chemical treatment with the chemical initiators has started between 14 and 17 hpf (hours post fertilization) and *gastrulation brain homeobox 2* (*gbx2*) expression was examined at 18 hpf. The effects of the chemicals on gbx2 expression were dependent on the time of exposure, with later time points (17 hpf) being less effective. Therefore, the timing of the chemical insult seems to be crucial for the toxicity of the chemicals (Wang et al., 2018).

##### MIE: Gsk3b Inactivation

Gsk3b kinase is constitutively active in resting cells and undergoes a rapid and transient inhibition in response to a number of external signals. Gsk3b activity is regulated by site-specific phosphorylation. Phosphorylation of Ser9 is the most common and important regulatory mechanism (Grimes and Jope, 2001; Luo, 2012). Gsk3b regulates diverse developmental events in the immature brain, including neurogenesis, neuronal migration, differentiation and survival in vertebrates (Kim and Kimmel, 2000). Human ortholog(s) of this gene are implicated in Alzheimer’s disease (Grassilli et al., 2014). Among the signaling proteins regulated by GSK3b are many transcription factors, including activator protein-1 (AP-1), cyclic AMP response element binding protein (CREB), heat shock factor-1 (HSF-1), nuclear factor of activated T cells (NFAT), MYC Proto-Oncogene (MYC), b-catenin, CCAAT/enhancer binding protein (C/EBP), and nuclear factor kappa-light-chain-enhancer of activated B cells (NFkB) (Grimes and Jope, 2001)

Essentiality—no experimental evidence found.

##### KER1: Gsk3b Inactivation Leads to Repression of gbx2 Expression

Wnt signaling is implicated in anteroposterior (AP) axis patterning and midbrain specification in animals. Gbx2 is one of the representative AP markers and is downregulated in activation of Wnt signal pathway, under Gsk3b inhibition. In zebrafish embryos, selective GSK3 inbition with BIO that activates Wnt signaling caused reduced expression of *gbx2* in the telencephalon (Wang et al., 2018), while in human ESC-derived cells GSKB inhibition with BIO was shown to downregulate GBX2 expression in a dose dependent manner (Kim et al., 2018). The role of Wnt signaling in *gbx2* regulation was confirmed via another Wnt signaling inhibitor (LiCl) that resulted in similar gene expression patterns of GBX2 in ESC-derived cells (Kim et al., 2018).

Biological plausibility—high. Inhibition of Gsk3b leads to activation of canonical Wnt signaling pathway (Grassilli et al., 2014), which is confirmed in many independent studies. Wnt signaling is implicated in AP axis patterning and midbrain specification in several vertebrate species, including humans. Gbx2 is one of the representative AP markers and is downregulated in activation of the Wnt signal pathway (Gsk3b inhibition) (Kim et al., 2018; Wang et al., 2018).

##### KE1: Repression of gbx2 Expression

During vertebrate brain development, the Gbx2 is expressed expressed in the forebrain (Wang et al., 2018). The genes encoding the Gbx-type homeodomain transcription factors have been identified in a variety of vertebrates, and are primarily implicated in the regulation of various aspects of vertebrate brain development (Nakayama et al., 2017). Gbx2 is involved in cerebellum development, iridophore differentiation, telencephalon regionalization and the anterior hindbrain development, where its role seem to be conserved at least in zebrafish and mice (Burroughs-Garcia et al., 2011). A number of studies have shown that Gbx2 represses many developmental regulatory genes during midbrain-hindbrain boundary development, including *forkhead box i1* (*foxi1*) (Simeone, 2000; Nakamura, 2001). Thus, Gbx2 may be a multifunctional transcriptional factor, although the mechanisms of the differential regulation of its activity during development are unknown (Nakayama et al., 2017).

Essentiality—no experimental evidence found.

##### KER2: Repression of gbx2 Expression Leads to Increased foxi1 Expression

The downregulation of foxi1 by Gbx2 in zebrafish embryos has been demonstrated in a transgenic fish line, using transient induction of *gbx2* and microarray analysis, later confirmed also by qPCR (Nakayama et al., 2017).

Biological plausibility—moderate. KER2 is supported based on a number of studies showing that Gbx2 represses many developmental regulatory genes (*foxi1*) (Simeone, 2000; Nakamura, 2001). Multifunctional nature of Gbx2 is still unknown and Gbx2 regulation of *foxi1* was measured indirectly with microarray analysis and later confirmed with qPCR. WISH (whole mount *in situ* hybridization) failed to confirm alterations of *foxi1* after *gbx2* expression but qPCR analysis showed immediate downregulation of *foxi1* (Nakayama et al., 2017).

##### KE2: Increased foxi1 Expression

Foxi1 is a transcription factor, involved in several processes, including animal organ development, epidermal cell fate specification, neuron development and the inner ear development, where it is involved in the induction of the otic-epibranchial progenitor domain (Hans et al., 2013).

Essentiality—high. When *foxi1* is knocked down, no expression of *SIX homeobox 1b* (*six1b*) is detected in the otocyst (Bricaud and Collazo, 2006). Therefore increase of *foxi1* expression is essential for downstream events in AOP to occur.

##### KER3: Increased foxi1 Expression Leads to Increased six1b Expression

When *foxi1* is knocked down, the ear anlagen is either entirely missing or greatly reduced (Solomon et al., 2003) and no expression of *six1b* is detectable (Bricaud and Collazo, 2006).

Biological plausibility—low. Foxi1 transcription factor regulates *six1b* and *EYA transcriptional coactivator and phosphatase 1* (*eya1*) gene expression (Bricaud and Collazo, 2006; Hans et al., 2013). Both are responsible for zebrafish inner ear development (Solomon et al., 2003). Regulation of *six1b* by Foxi1 was confirmed with loss-of-function experiment, but connection could be secondary to the overall absence of the otic placode attributable to *foxi1* loss-of-function (Bricaud and Collazo, 2006).

##### KE3: Increased six1b Expression

Six1b is predicted to have DNA-binding transcription factor activity, RNA polymerase II-specific and RNA polymerase II cis-regulatory region sequence-specific DNA binding activity. It is involved in several processes, including muscle organ development, nervous system development and regulation of skeletal muscle cell proliferation. *Six1b* is a Member of the *Pax–Six1b–Eya–Dach* (*paired box–sine oculis homeobox–eyes absent–dachshund*) gene regulatory network, involved in the development of numerous organs and tissues (Bessarab et al., 2004; Bricaud and Collazo, 2006). It has been proposed to play an important role in inner ear development. Six1b promotes hair cell fate and, conversely, inhibits neuronal fate by differentially affecting cell proliferation and cell death in these lineages. Gain/loss-of-function experiment results indicate that when *six1b* is overexpressed, not only are fewer neural progenitors formed, but many of these progenitors do not go on to differentiate into neurons (Bricaud and Collazo, 2006).

Essentiality—moderate. Six1b promotes hair cell fate and, conversely, inhibits neuronal fate by differentially affecting cell proliferation and cell death in these lineages. *Six1b* gain/loss-of-function experiment results indicate that in both cases normal development of inner ear is affected (KE5) (Bricaud and Collazo, 2006).

##### KER4: Increased six1b Expression Leads to Inhibited eya1 Expression

Eya1 and Six1b together with the Dach protein directly interact to form a functional transcription factor. In this complex, the DNA binding function is provided by the Six protein, while Eya mediates transcriptional activation and Dach proteins appear to function as cofactors (López-Ríos et al., 2003). *Six1b* gain-of-function experiment results showed that overexpression of *six1b* in zebrafish developing inner ear reduced the expression of *eya1* (Bricaud and Collazo, 2006).

Biological plausibility—low. Although there is evidence to support the relationship, there is still too little known about interactions and regulation of both entities, as it is thought to act in a complex like transcription factor (López-Ríos et al., 2003). In addition, interactions between Six1b and Eya1 in zebrafish and mice are not conserved, according to several studies (Xu et al., 1999; Li et al., 2003; Zheng et al., 2003).

##### KE4: Inhibited eya1 Expression

Eya1 is predicted to have protein tyrosine phosphatase activity and is involved in adenohypophysis development, otic vesicle morphogenesis, and otolith development in both vertebrates and invertebrates. In zebrafish, the *eya1* gene is widely expressed in placode-derived sensory organs during embryogenesis. Eya1 function appears to be primarily required for survival of sensory hair cells in the developing ear and lateral line neuromasts (Kozlowski et al., 2005).

Essentiality—no experimental evidence found.

##### KER5: Inhibited eya1 Expression Leads to Increased Cell Death

Zebrafish Eya1 has a role in development of the cristae, statoacoustic ganglia, and lateral line system. An *eya1* disrupted zebrafish mutant (named dog-eared) features premature apoptosis in precursors to these structures. Because of the large number of apoptotic cells observed within the otic vesicle of the mutants, it has been proposed that Eya1 could act as a suppressor of apoptosis (Kozlowski et al., 2005). In mammals EYA1 dephosphorylates histone variant H2AX and thereby affects DNA repair and cell survival (Cook et al., 2009).

Biological plausibility—high. Eya1 role in regulating cell death within developing otic vesicles is well established in vertebrates (Xu et al., 1999; Cook et al., 2009). Zebrafish *dog-eared* mutants are one of the models for human deafness disorders. *Dog-eared* zebrafish ear phenotype shows sensory and non-sensory defects (Whitfield, 2002).

##### KE5: Increased Cell Death (https://aopwiki.org/events/1825)

Essentiality—high. While not the sole contributor to altered inner ear development, cell death is one of the key players in normal development of sensory organs (Whitfield, 2002; Kozlowski et al., 2005).

##### KER6: Increased Cell Death Leads to Altered Inner Ear Development

Increased levels of apoptosis occur in the migrating primordia of the posterior lateral line in the dog-eared zebrafish embryo mutants, resulting in smaller otic vesicles (Kozlowski et al., 2005). The lateral line placodes of fishes and amphibians also give rise to hair cells and supporting cells, which form small mechanosensory organs (neuromasts) distributed in lines along the body surface and involved in the detection of water movements. They also produce the sensory neurons innervating these receptor organs (Schlosser, 2014). After *six1b* or *eya1* loss of function, the numbers of sensory receptors and neurons in the sense organs and ganglia derived from the olfactory, otic, lateral line, profundal/trigeminal, and epibranchial placodes are reduced, and only small, malformed sensory organs develop that are abnormally patterned and functionally deficient (Schlosser, 2014).

Biological plausibility—high. There is a number of consistent studies that support the relationship of increased cell death during the development and defect in the development of the inner ear (Whitfield et al., 1996; Kozlowski et al., 2005; Schlosser, 2014).

##### KE6: Altered Inner Ear Development

Zebrafish do not possess outer or middle ears, but have a fairly typical vertebrate inner ear, the normal development and anatomy of which has been extensively described (Haddon and Lewis, 1996; Bang et al., 2001). Although the zebrafish ear does not contain a specialized hearing organ—there is no equivalent of the mammalian cochlea—many features are conserved with other vertebrate species (Whitfield et al., 2002). The mature inner ear, found in all jawed vertebrates, has two functions: It serves as an auditory system, which detects sound waves, and as a vestibular system, which detects linear and angular accelerations, enabling the organism to maintain balance (Whitfield et al., 1996).

Essentiality—no experimental evidence found.

##### KER7: Altered Inner Ear Development Leads to Reduced Hearing

Mutations in several genes connected to development of inner ear affect morphology and patterning of the inner ear epithelium, including formation of the semicircular canals and development of sensory patches (maculae and cristae). Dog-eared mutants show abnormal development of semicircular canals and lack cristae within the ear (Kozlowski et al., 2005). Zebrafish mutant embryos with defects of the inner ear fail to balance correctly, and may swim on their sides, upside down, or in circles (Whitfield et al., 1996).

Biological plausibility—high. The inner ear is the vertebrate organ of hearing and balance (Whitfield, 2002). Several zebrafish mutants for studying development of inner ear like *dog-eared* or *van gogh* show defects in inner ear development and irregular swimming patterns (Whitfield et al., 1996).

##### KE7: Reduced Hearing

Hearing refers to the ability to perceive sound vibrations propagated as pressure changes through a medium such as air or water. Reduced hearing in the context of this key event can refer to reduction in the perceived volume of a sound relative to the amplitude of sound waves. Reduced hearing may also refer to a reduced range of frequencies that can be perceived. Zebrafish serves as a model organism for hearing and deafness. Zebrafish mutant embryos fail to balance correctly, and may swim on their sides, upside down, or in circles (Whitfield et al., 1996).

Essentiality—no experimental evidence found.

##### KER8: Reduced Hearing Leads to Increased Mortality

Although we are not aware of any studies directly looking into increased mortality as a results of reduced hearing ability in fish, it is known that hearing ability is very important for fish, with roles in everything from reproduction to swimming (Kasumyan, 2009). It is therefore very likely that a reduction in the vital sensory ability would negatively affect fish survival.

Biological plausibility—high. Impaired hearing can result in changes in ecologically relevant endpoints, such as predator avoidance and prey capture. Therefore, it can be assumed that an effect on hearing could reduce young of year survival. The relationship is well accepted and corresponds to the logic of nature.

##### AO: Increased Mortality (https://aopwiki.org/events/351) Overall Assessment of the AOP 410

An overall assessment of this AOP shows that there is low to moderate biological plausibility to suggest a qualitative link between the inactivation of Gsk3b to the KE4-cell death within developing inner ear and high evidence linking KE5 to increased mortality (AO). Biological plausibility is considered moderate because there is ample evidence from gain- and loss- of function experiments and knock out animal models that support the relationships between key events which are consistent with current biological knowledge, but there is mostly indirect evidence linking KEs on molecular level. KEs on molecular level have some uncertainties like *foxi1* loss of function experiment resulting in no expression of *six1b* in otic placode (due to absence of otic placode) and inconsistencies across species (zebrafish, mouse). No empirical evidence in the form of dose response, time concordance and incidence concordance was found for the KERs of the AOP, and only little evidence for the essentiality of the KEs. The summarized information on KER biological plausibility, KE essentiality and KE observation methods of AOP 410 can be found in Tables 3–5.

## Discussion

In this study we evaluated the suitability of causal toxicological networks (CTNs) for AOP development. We have developed a semi-automatic pipeline and scripts that take a CTN in BEL format, remove unneeded node and connection annotations and add new functional ones, then reduce the network to a size similar to an AOP network (Pollesch et al., 2019). We performed these operations on a recently developed zebrafish developmental neurotoxicity network as a case study (Li et al., 2021). The network was then separated into subnetworks, based on different starting points (candidate MIEs of different types) and endpoints (pathologies), with a size suitable for close visual inspection. Finally, we separated the subnetworks into a set of candidate AOPs related to zebrafish neurotoxicity. Both the pipeline and the set of candidate AOPs can be found in the Supplementary Material and will hopefully be used for further endeavors in neurotoxicity AOP development.

The second contribution of the study is the development of two new AOPs, one centered on reduced eye size and decrease visual function (AOP 399) and another on alterations in inner ear development and decreased hearing (AOP 410) of the zebrafish. Both newly developed AOPs are available in the AOP-Wiki and have added to the very few neurotoxicity AOPs already there. Neurotoxicity AOPs are especially difficult to develop, as the brain is an extremely complex organ, comprised of a variety of highly specialized neural and glial cell types with diverse cellular functions. This implies the existence of a potentially large number of AOPs and the great need to develop more as soon as possible (Bal-price et al., 2015). As far as the WoE analysis of AOPs 399 and 410, the biological plausibility for most KERs in both AOPs is high, with a few KERs where plausibility is moderate/low. The evidence for the essentiality of the KEs is mostly missing. This means that AOP 399 and AOP 410 require further improvements before regulatory use and that we should probably still refer to them as plausible AOPs. However, even if not immediately useful for regulation purposes, plausible AOPs lacking some evidence are useful for identification of missing knowledge and targeted design of new experiments that can fill the identified gaps (Perkins et al., 2015).

But, perhaps most importantly, while developing AOPs 399 and 410, we have also encountered some advantages and challenges that are specific to development of AOPs from CTNs. A very important advantage is that, because of the nature of the design/development of CTNs (Li et al., 2020), which is based on direct experimental evidence connecting the nodes in the network, the biological plausibility of the KERs of the derived AOPs is mostly high/moderate. Since biological plausibility is the most important characteristic of AOPs (OECD, 2014; Fay et al., 2017), this makes CTNs an excellent starting point for AOP development. On the other hand, because of the same reason, much less evidence can be found for KE essentiality and even less empirical evidence in the form of dose response, time concordance and incidence concordance, which are essential for qAOP development. Nevertheless, the AOPs developed from CTNs are consistent with the core principles of AOP development (Villeneuve et al., 2014a).

In our results section we describe all the details that came up when developing the AOP, which will hopefully be of use for future AOP development, either with the same zebrafish neurotoxicity network or with another CTN. As our described development of AOP 410 suggests, the network manipulations necessary to come to a smaller, easier to handle network that can be used as AOP starting points, can also cause some nodes and evidence to disappear. Therefore, although a CTN may make it easier to construct a new AOP, for some KE and KERs it is still necessary to add additional evidence and handle WoE assessment with utmost care. In our case, in the original network cell death and otic vesicle formation nodes were not causally connected, probably because not enough literature was added to the initial network or the computational pipeline did not detect causal dependence of entities in it. It is therefore worth noting that although a lot of information is stored in CTNs, they are by no means complete. When comparing the number of references in the CTN-based candidate AOPs that we started from to the number of references in the final AOPs 399 and 410, we can see that only ca. 20 % were already in the CTN and ca. 80 % were added through the search for additional evidence. However, the original 20% were a very good starting point, as many of the additional references were added based on searching for scientific studies cites or citing these original 20 %.

As we (the authors of this paper) have little experience in developing AOPs using other strategies, it is difficult to assess the practical advantages or disadvantages of the proposed approach, either with respect of time investment or expertise needed. It is in our opinion certain that developing sets of candidate adverse outcome pathways that are all backed by sound evidence can be done very quickly using the proposed methodology. From an experimental point of view, the candidate AOPs represent working hypotheses, which can help guide targeted experiments that add to our toxicological knowledge base. However, choosing among this candidate set, developing the AOP and performing the WoE will still require the same amount of time and expertise as with any other approach that we are aware of, as the evidence available in the CTN cannot be taken for granted, but needs to be reevaluated for each new AOP. It has been argued that AOP networks, with their increased complexity have advantages in representing the toxic effects of chemicals (Knapen et al., 2018; Villeneuve et al., 2018), so the question arises why not use CTNs to directly develop AOP networks, instead of AOPs, as we did here. However, AOP networks are defined as networks of individual AOPs, joined at shared KEs. The focus is therefore on KEs and KERs and the evidence supporting them. Once the weight of evidence analysis is performed, AOP networks can be easily put together from the AOP-Wiki, as demonstrated recently (Ankley et al., 2020).

When we started the development of the new AOPs, there were no AOPs on the AOP-wiki that would feature the KEs related to eye size and ear development. We were thus pleasantly surprised when soon afterwards the reduced eye size KE appeared and we could link the evidence we found to a collection of evidence already there (but found from different sources). Surprisingly, although we purposefully avoided developing AOPs that would already be present in the AOP-Wiki, for both developed AOPs we ended up connecting to other KE in other AOPs. For AOP 399 it was because AOP 364 (entitled Thyroperoxidase inhibition leading to altered visual function via decreased eye size) added the same KE only a couple of weeks before we did. For AOP 410 it was due to the lack of evidence for reduced eye size caused by *gbx2* expression inhibition that we moved the development towards the inner ear and the upstream MIE of Gsk3b inactivation, which was also already present in the AOP 298 (entitled Chronic ROS leading to human treatment-resistant gastric cancer). With more AOPs being developed, we expect that more and more KEs and KERs will be shared among them and that the AOP world will quite soon become a large AOP network. This should in turn speed up the development of new AOPs.

## Data Availability

The original contributions presented in the study are included in the article/Supplementary Material, further inquiries can be directed to the corresponding author.
