# Supplementary material for: From Causal Networks to Adverse Outcome Pathways: A Developmental Neurotoxicity Case Study
Source: Front Toxicol. 2022 Mar 7;4:815754. doi: 10.3389/ftox.2022.815754 (PMC8915909; doi:10.3389/ftox.2022.815754)
Supplement: Supplementary file 1 [file DataSheet1.docx]

Supplementary Material

# Supplementary Figures and Tables

## Supplementary Figures


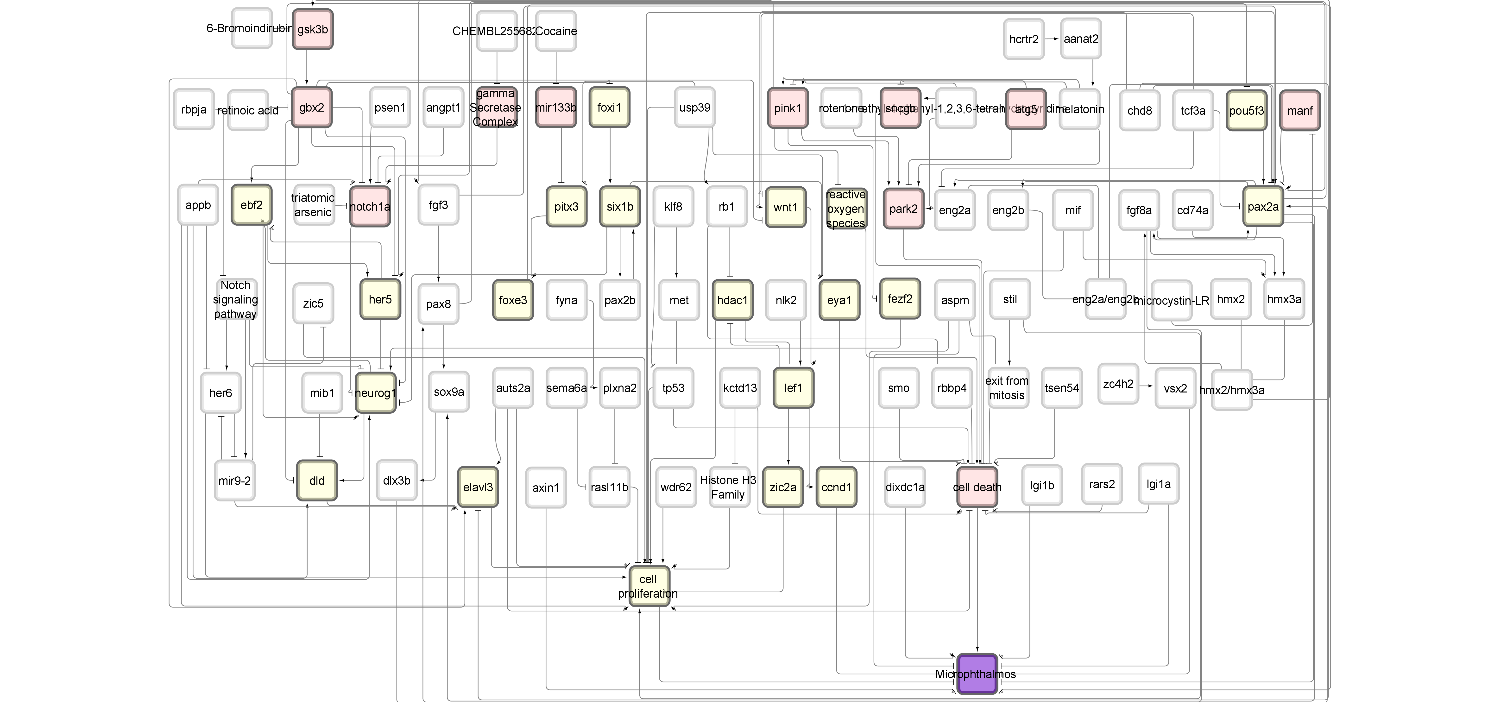


**Supplementary Figure 1.** The whole reduced NTOX network where the start nodes are small molecules (red) and the final node is microphthalmos (purple). All other nodes that are part of the candidate AOPs are marked in yellow.


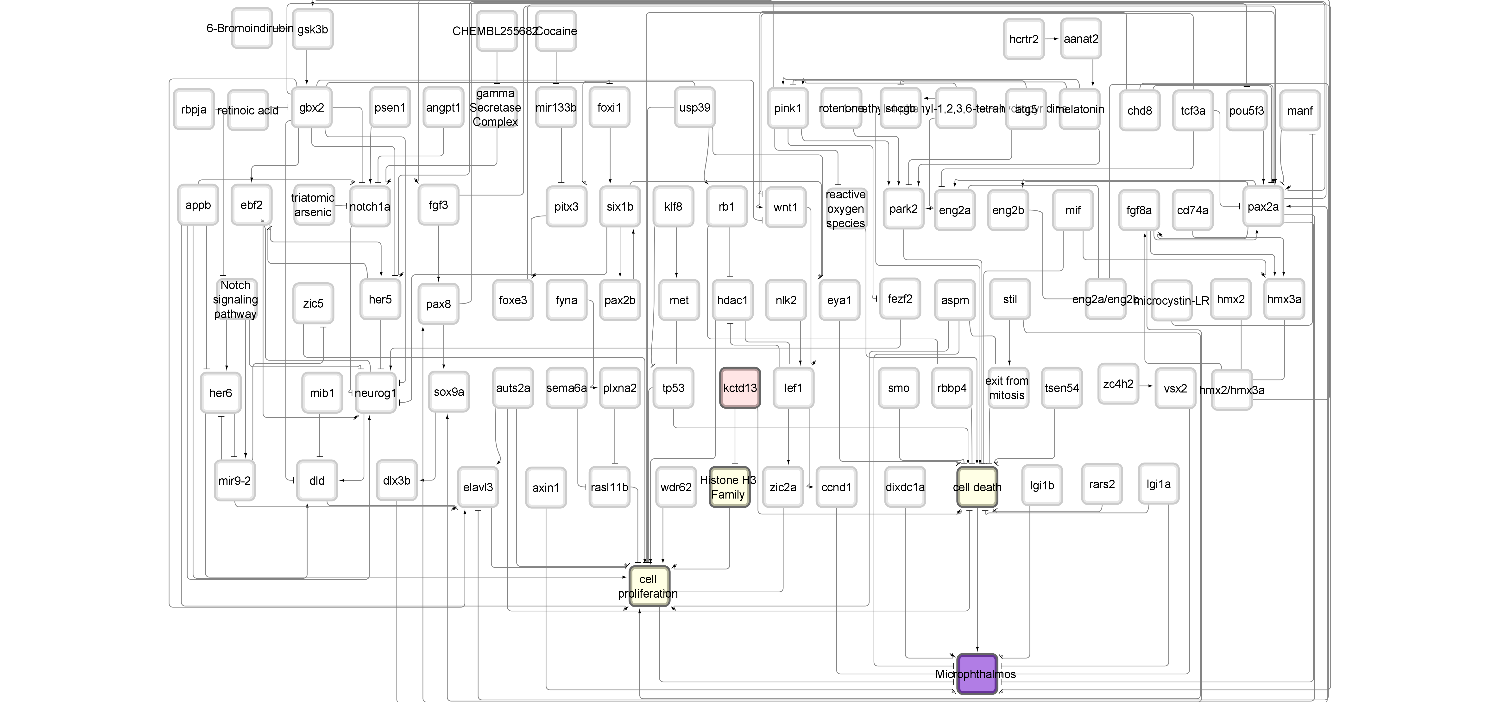


**Supplementary Figure 2.** The whole reduced NTOX network where the start nodes are transporters or channels (red) and the final node is microphthalmos (purple). All other nodes that are part of the candidate AOPs are marked in yellow.


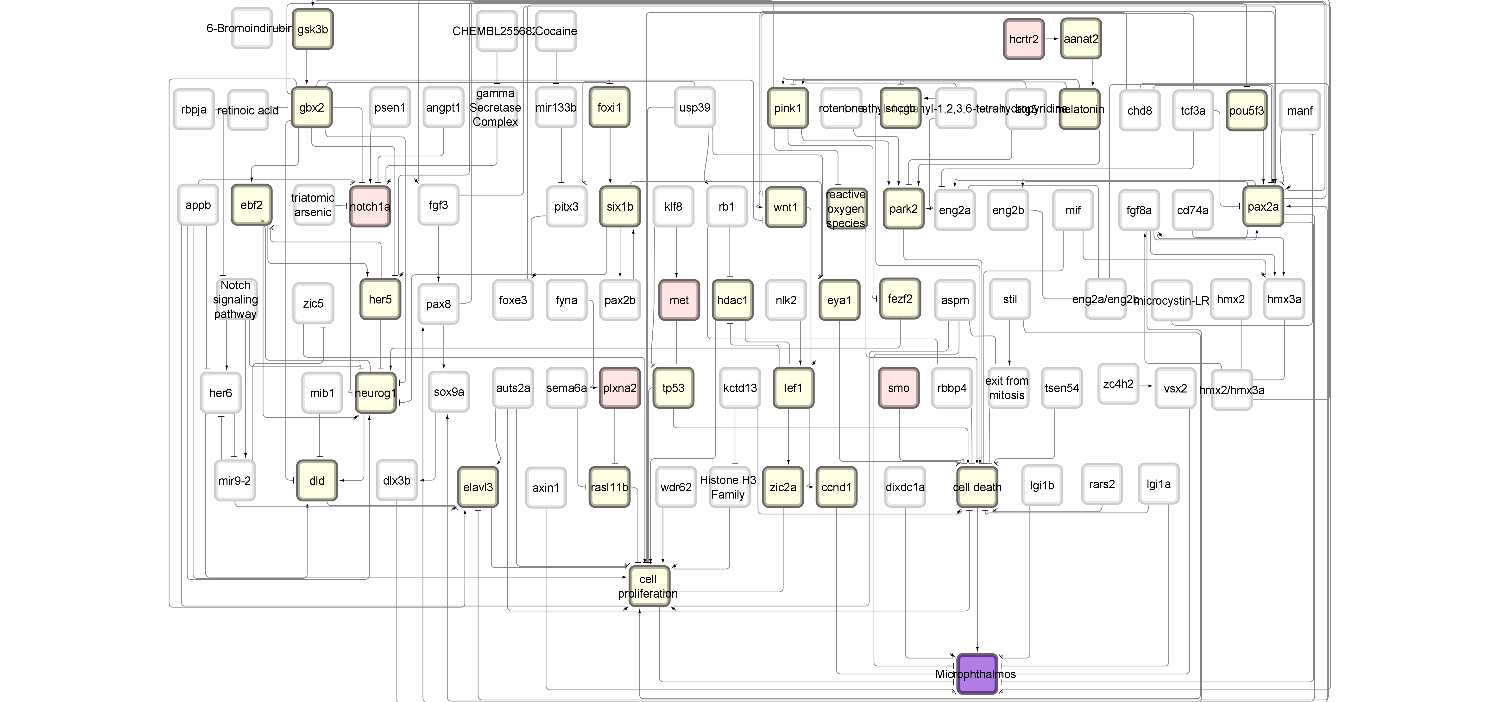


**Supplementary Figure 3**. The whole reduced NTOX network where the start nodes are receptors (red) and the final node is microphthalmos (purple). All other nodes that are part of the candidate AOPs are marked in yellow.

## Supplementary Tables

**Supplementary Table 1.** Examples of modifications for all node definition types and all reactions during the simplification of the BEL formalism network. ‘Level’ defines whether the action was performed on the node name level or the reaction. For each BEL function, examples are given comparing the BEL formalism (column 3) with the simplified abstracted formalism.

| Level | BEL function | BEL formalism | ABSTRACTED |
| --- | --- | --- | --- |
| node | abundance | a(CHEBI:acrylamide) | acrylamide |
| node | rnaAbundance | r(ZFIN:gata4) | gata4 |
| node | proteinAbundance | p(ZFIN:gata4)  p(ZFIN:gata4,pmod(P)) | gata4  gata4 |
| node | microRNAAbundance | m(ZFIN:mir182) | mir182 |
| node | molecularActivity | act(p(ZFIN:actn2b))  act(p(SFAM:"MAPK Erk1/2 Family")) | actn2b  MAPK Erk1/2 Family |
| node | biologicalProcess | bp(GOBP:"neuron differentiation") | neuron differentiation |
| node | complexAbundance /  compositeAbundance | complex(p(ZFIN:nfatc4),p(ZFIN:gata4)) | nfatc4/gata4 |
| node | pathology | path(MESHD:"Heart Failure") | Heart Failure |
| EDGE | actsIn() | X actsIn Y | actsIn |
| EDGE | decreases() | X decreases Y | inhibition |
| EDGE | directlyDecreases() | directlyDecreases | inhibition |
| EDGE | directlyIncreases() | directlyIncreases | activation |
| EDGE | hasComponent() | hasComponent | binding |
| EDGE | hasModification() | hasModification | hasModification |
| EDGE | includes() | includes | binding |
| EDGE | increases() | increases | activation |
| EDGE | interacts with() | interacts with | binding |
| EDGE | translatedTo() | translatedTo | translatedTo |
